# The immune mechanisms of the urinary tract against infections

**DOI:** 10.3389/fcimb.2025.1540149

**Published:** 2025-04-16

**Authors:** Yilin Hou, Zhuoxuan Lv, Quanjie Hu, Aisong Zhu, Hongxia Niu

**Affiliations:** ^1^ School of Basic Medical Science, Zhejiang Chinese Medical University, Hangzhou, China; ^2^ Key Laboratory of Blood-Stasis-Toxin Syndrome, Zhejiang Chinese Medical University, Hangzhou, China

**Keywords:** urinary tract infection, Uropathogenic Escherichia coli, immune mechanism, urothelial cell, adaptive immunity, innate immunity

## Abstract

Urinary tract infection (UTI), a common clinical infectious disease, is marked by high incidence and frequent recurrence. Recurrent UTIs can cause severe complications, negatively affecting health. The emergence and spread of drug-resistant bacteria present significant challenges to UTI treatment. This article systematically reviews the key immune mechanisms in the body’s defense against UTI pathogens. It discusses various immune response components, such as the urinary tract mucosal epithelium, neutrophils, macrophages, dendritic cells, mast cells, innate lymphocytes, T cells, and B cells, with the aim of providing insights for future UTI research.

## 1 Introduction

Urinary tract infection (UTI) is a prevalent bacterial infectious disease, affecting around 150 million people globally each year (Foxman, 2014). UTI not only has a notably high incidence but also tends to recur. Repeated episodes may lead to severe complications such as pyelonephritis, sepsis, and renal damage (Chugh et al., 2020), which pose significant threats to patients’ health and lives. Reports suggest that 20%-30% of patients with a first infection will experience reinfection (Foxman et al., 2000). Moreover, the increasing prevalence of antibiotic-resistant uropathogens and their transmission in clinical settings complicate UTI treatment, especially in recurrent cases (Halbay, 2023).

The relationship between immune function and recurrent infections is critical. UTIs arise from an imbalance between bacterial virulence and host immune defenses, and alterations in these host mechanisms act as risk factors for UTI onset (Garcia et al., 2018). Recent advances in understanding the urinary immune system and its anti-infection mechanisms has provided significant insights, uncovering new pathways contributing to UTI development. This article systematically reviews the cells and molecules involved in urinary immune defense, using the infection process of Uropathogenic Escherichia coli (UPEC) as a case study to illustrate the mechanisms of urinary anti-infection immunity and recent research advances.

## 2 Barrier and defensive functions of urothelial mucosal epithelial cells

**Figure 1:**
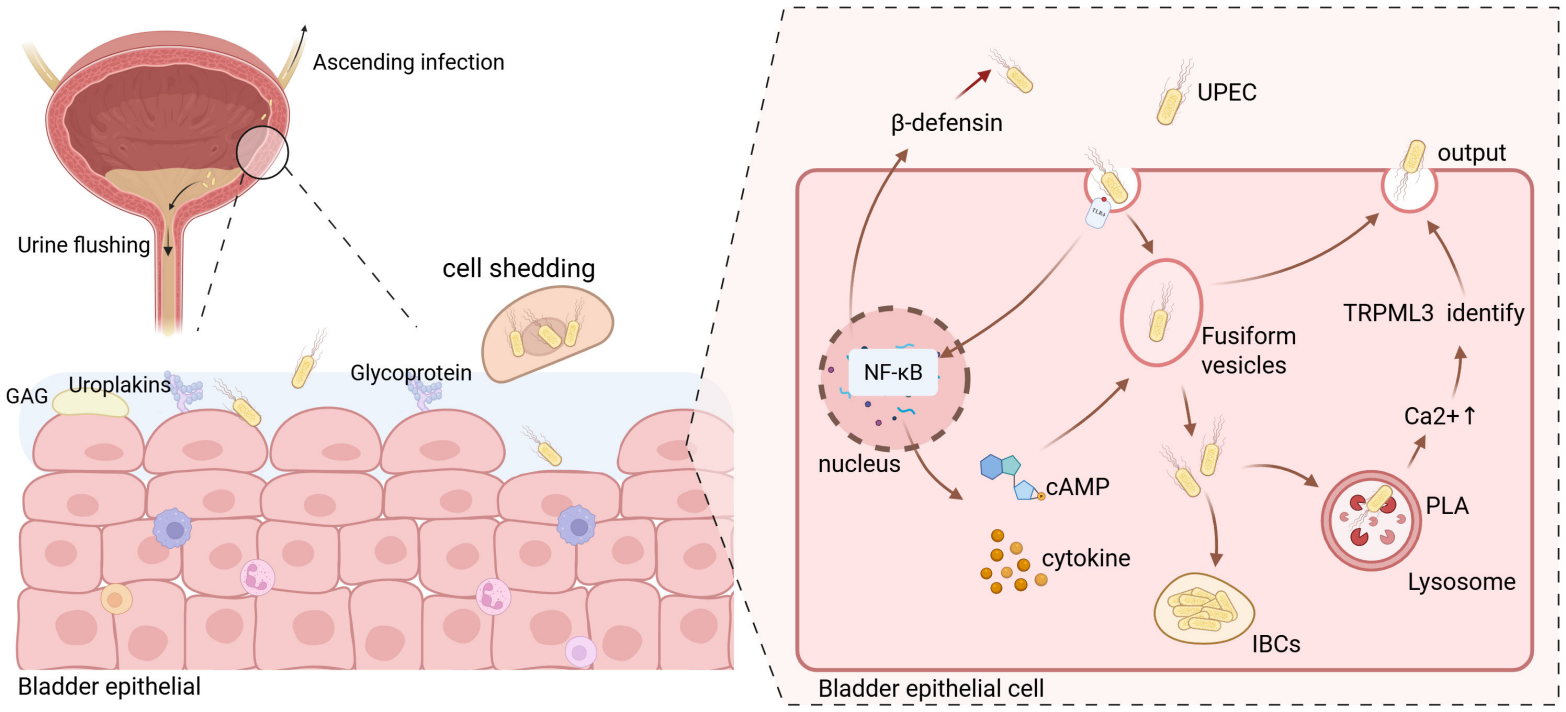
Schematic illustration of Uropathogenic Escherichia coli (UPEC) invasion into bladder epithelial cells. Due to the relatively short length of the female urinary tract, UPEC typically enters the bladder through the urethra and attempts to invade bladder epithelial cells. The urine accumulated in the bladder serves to flush out most bacteria; however, a small fraction of UPEC that adheres to the surface of the bladder epithelial cells proceeds to invade further and may even cause ascending infections. UPEC enters the bladder epithelial cells via endocytosis. Simultaneously, the toll-like receptors (TLRs) on the cell surface transmits signals that activate the nuclear factor kappa-B (NF-κB) signaling pathway. This activation prompts the release of a substantial amount of cytokines and cyclic adenosine monophosphate (cAMP). The produced β-defensin is then secreted into the extracellular space to counteract the invasion of UPEC. Moreover, the released cAMP promotes the exocytosis of vesicles containing UPEC. The cytokines generated primarily combat the invasion of UPEC by recruiting immune cells. Meanwhile, a portion of UPEC within the vesicles can breach the vesicle membrane and enter the cytoplasm. The majority of these intracellular bacteria will be recognized by lysosomes and ultimately be excreted out of the cells. However, a minuscule number of them are capable of forming intracellular bacterial communities (IBCs) within the cells. Created with BioRender.com.

Bladder epithelial tissues and cells are crucial for maintaining bladder function and defending against pathogenic invasion (**Figure 1**). They act as a barrier against toxic substances in urine and combat bacteria via various immune mechanisms (Marshall et al., 2019; Garcia et al., 2018). The bladder epithelium is a stratified transitional structure with three layers: basal cells, intermediate cells, and umbrella cells (bladder epithelial cells, BECs). Umbrella cells maintain the impermeability and high-resistance barrier of the urinary mucosal surface through tight junctions (Jaimes-Parra et al., 2015; Winder et al., 2014; Osborn and Kurzrock, 2014). The surface of bladder epithelial cells is coated with a mucosal layer of highly negatively charged proteoglycans and glycosaminoglycans, the glycosaminoglycan (GAG) layer, which protects the epithelium from toxic damage (Terlizzi et al., 2017). The bladder epithelium is also covered with uroplakin protein surface plaques (uroplakins, Ups) (Wu et al., 2017; Hill, 2015). Uroplakin proteins are primary receptors for UPEC adhesion, and their glycosylation changes are linked to urinary epithelium pathologies like UTIs and interstitial cystitis (Taganna et al., 2011).

Bladder epithelial cells show morphological plasticity, and fusiform vesicle endocytosis and exocytosis regulate the bladder surface area during urine accumulation (Grasso and Calderón, 2013). During urine voiding, bladder epithelial cell membranes invaginate to form fusiform vesicles that internalize UPEC, leading to bacterial endocytosis (Wu et al., 2017). The fusiform vesicle membrane is similar to the cell membrane and rich in Toll-like receptor 4 (TLR4). TLR4 binding with UPEC activates multiple signaling pathways, including nuclear factor kappa - B (NF - κB), promoting cytokine, inflammatory mediator, and antimicrobial peptide (AMPs) release, exerting a defensive effect. For example, increased intracellular cyclic adenosine monophosphate (cAMP) levels cause Rab27b + fusiform vesicle exocytosis, releasing bacteria into the bladder lumen (Miao et al., 2016). Research shows that within 24 hours of bacterial invasion, over 90% of intracellular bacteria are expelled, demonstrating bladder epithelial cell anti - infection ability (Bishop et al., 2007). However, UPEC expressing phospholipase A (PLA) can penetrate Rab27b + vesicles and enter the cytoplasm, where a few bacteria can replicate to form intracellular bacterial communities (IBCs) (Pang et al., 2022). IBCs allow UPEC to colonize bladder epithelial cells and initiate disease, enhancing antibiotic resistance. After entering bladder epithelial cells, UPEC is recognized by autophagosomes and sent to lysosomes for degradation (Wu et al., 2017). But UPEC can neutralize lysosome pH and disrupt degradation. Abnormal lysosomes are sensed by transient receptor potential mucolipin 3 (TRPML 3), releasing Ca2 + from lysosomes into the cytoplasm and facilitating expulsion of dysfunctional lysosomes with bacteria from the cell.

Bladder epithelial cells release antimicrobial peptides limit pathogen survival in early infection. For example, β - defensin 1 expression increases after TLR4 activation (Hickling et al., 2013). Uromodulin (UrM) can competitively bind to UPEC type 1 fimbriae, preventing UPEC adhesion to uroepithelial cells (Bowyer et al., 2022). If bladder epithelial cell defenses fail to eliminate intracellular bacteria, infected superficial cells undergo caspase - 3 and caspase - 8 - dependent apoptosis, shedding to reduce bacterial load. Basal and intermediate cells then proliferate and regenerate superficial cells. But this exposes intermediate cells to pathogen - containing urine, risking deeper tissue infection and damage.

Uncleared UPEC in the bladder can ascend to the kidneys, causing upper UTI. Renal units consist of principal cells (PCs) and intercalated cells (ICs), with ICs subdivided into type A, type B, and non - type A non - type B (Roy et al., 2015). UPEC adheres to type A intercalated cells, entering via complement internalization or cellular lipid rafts (Neal et al., 2014; Chassin et al., 2006). Renal epithelial cells release inflammatory chemokines and cytokines related to neutrophils, monocytes, or macrophages through pattern recognition receptors, recruiting corresponding immune cells to combat UPEC (Schwartz et al., 2023). Infected intercalated cells increase phagosome maturation - related gene expression, promoting UPEC phagocytosis (Saxena et al., 2021). Intercalated cells also release AMPs, cytokines, and protons into the urine to counteract pathogens (Becknell et al., 2015; Saxena et al., 2018, Saxena et al., 2019). Intercalated cells are associated with UPEC susceptibility. Carbonic anhydrase 2 (CA2) is important for intercalated cell function. Mice lacking CA2 have 50% fewer intercalated cells and impaired UPEC clearance, and transplanting their kidneys into wild - type mice increases pyelonephritis risk (John et al., 2020; Hains et al., 2014). This suggests intercalated cells may prevent renal pyelonephritis, though mechanisms need further study.

The renal tissue extracellular microenvironment and hormone levels affect antibacterial defense. High medullary sodium concentrations enhance kidney antibacterial capacity (Schwartz et al., 2023). During pyelonephritis, the medulla’s hyperosmotic environment promotes monocyte chemotactic protein - 1 (MCP - 1) production by nuclear factor activated T cell 5 (NFAT5) in renal tubular cells, recruiting CD14 + mononuclear phagocytes (MNP) to the medulla (Schwartz et al., 2023). MNP differentiate into M1 macrophages to eliminate UPEC. Medullary epithelial cells and macrophages secrete granulocyte chemokines like IL - 8 and TNF - α, recruiting neutrophils (Naskar and Choi, 2024). Additionally, uromodulin (UrM) released into the renal tubule lumen can competitively bind to type 1 pili expressed by uropathogenic Escherichia coli (UPEC), thereby preventing UPEC adherence to epithelial cells (Mercado-Evans et al., 2025). Changes in the medulla’s sodium concentration gradient can cause abnormal cytokine production, reducing MNP recruitment and increasing pyelonephritis risk (Berry et al., 2017). This shows the renal medulla microenvironment is crucial for immune defense during UTIs. Hormones like antidiuretic hormone, insulin, and sex hormones also play roles in kidney resistance to UPEC infections. Pharmacological antagonism of arginine vasopressin receptor 2 (AVPR2) on renal principal cells stimulates cytokine production and UPEC clearance in infected mice (Zetter et al., 2019). Moreover, insulin might be related to the expression of certain antimicrobial peptides. In the kidneys of rodents with type 1 and type 2 diabetes, the level of Bd1, which encodes the antimicrobial peptide β - defensin 1, is lower compared to that in the kidneys of healthy animals (Froy et al., 2007). A study shows that local insulin receptor deficiency in the bladder urothelium can promote the occurrence of UTI by increasing barrier permeability and inhibiting antimicrobial peptides (Schwartz et al., 2024). In addition, research data indicate that male mice are more susceptible to pyelonephritis and renal abscesses than female mice. Castration of male mice or inhibition of androgen receptor signaling can decrease the risk of urinary tract infections (Hreha et al., 2020a, Hreha et al., 2020b; Olson et al., 2016; Olson et al., 2018). In another study, within 24 hours post - infection, female C57BL/6 and C3H/HeN mice exhibited a more robust cytokine response and a more rapid recruitment of immune cells within the bladder when compared to male mice. This characteristic allows normal female mice to efficiently clear the infectious pathogens present in the bladder within 7 days (Hreha et al., 2024). Although male mice have a higher number of myeloid cells in the circulatory system than female mice, testosterone can attenuate the function of renal neutrophils by impeding neutrophil maturation. This leads to inadequate infiltration of neutrophils in the kidneys of androgen - exposed hosts, rendering them unable to fully control the infection (Hreha et al., 2024). However, at present, the specific mechanism through which androgens inhibit neutrophil maturation remains obscure. Furthermore, it remains to be determined whether androgens can influence the course of urinary tract infections (UTIs) via other pathways.

## 3 The anti-infection mechanisms of innate immune cells and molecules

### 3.1 Neutrophils

**Figure 2:**
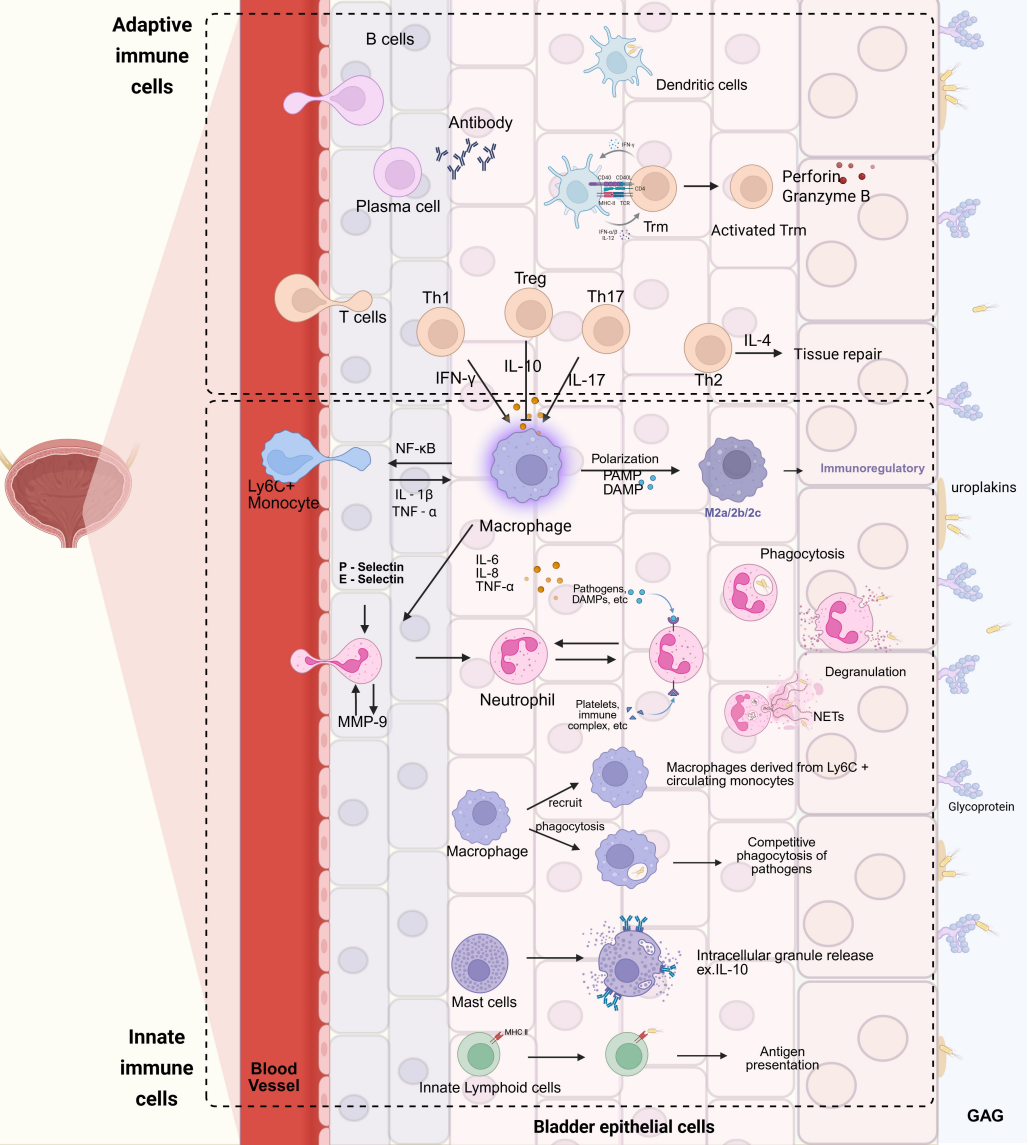
Schematic representation of the immune response to bacterial invasion in the urinary tract. Uropathogenic Escherichia coli (UPEC) initiates the process by penetrating the glycosaminoglycan (GAG) layer and adhering to uroplakins (Ups) on the surface of urinary tract epithelial cells. Upon invasion of the epithelial cells, toll-like receptors (TLRs) are activated, triggering a cascade of cellular immune responses. Neutrophils, in response to chemotactic signals mediated by P-selectin and E-selectin, extravasate from blood vessels and release matrix metalloproteinase-9 (MMP-9), which further enhances neutrophil extravasation and migration to the site of infection. Ly6C+ monocytes, under the stimulation of interleukin-1β (IL-1β) and tumor necrosis factor-α (TNF-α), undergo proliferation and differentiate into macrophages. Subsequently, in the presence of pathogen-associated molecular patterns (PAMPs) and damage-associated molecular patterns (DAMPs), these monocytes can polarize into M2 macrophages. T cells, upon activation, differentiate into distinct subsets, including T helper 1 (Th1), T helper 2 (Th2), T helper 17 (Th17), and regulatory T cells (Tregs). Th17, Treg, and Th1 cells modulate macrophage functions by secreting interleukin-17 (IL-17), interleukin-10 (IL-10), and interferon-γ (IFN-γ), respectively, while Th2 cells promote tissue repair through the release of interleukin-4 (IL-4). Activated B cells differentiate into effector B cells, namely plasma cells, which secrete antibodies to combat pathogen invasion. In addition to the immune cells recruited from the peripheral blood, tissue-resident cells also play pivotal roles. Macrophages residing in tissues can phagocytize pathogens and recruit Ly6C+ macrophages derived from monocytes. Dendritic cells are capable of phagocytosing pathogens and presenting antigens to initiate adaptive immune responses. Innate-like lymphocytes, through MHC class II molecules on their surface, recognize pathogens and participate in antigen presentation. Mast cells release intracellular granules, such as interleukin-10 (IL-10), to counteract UPEC infection. Tissue-resident memory T cells (Trm), once activated, actively engage in the immune response, collaborating with other immune cells to resist UPEC invasion. Created with BioRender.com.

Upon detecting pathogen invasion, urinary tract epithelial cells promptly mobilize resident tissue cells or circulating immune cells to the infection site via signaling pathways for bacterial clearance (**Figure 2**). Neutrophils are the primary innate immune cells in fighting urinary tract infections and are vital for controlling early UPEC-induced infections (Hayes and Abraham, 2016; Ashkar et al., 2008). Neutrophil recruitment can be initiated by inflammatory mediators from leukocytes in the infected tissue or cytokines generated through intracellular signaling activated by Toll - like receptor 4 (TLR4) on bladder epithelial cell surfaces and the cytoplasmic Toll/IL - 1 receptor homology domain (TIR) (Yu et al., 2019; Hannan et al., 2010; Billips et al., 2007). The released cytokines like IL - 6, IL - 8, and TNF - α show elevated expression levels correlating with bacterial clearance. Notably, IL - 8 is the main chemokine directing neutrophils to the infection site (Bowyer et al., 2022). After infection, bladder epithelial cells quickly upregulate P - selectin and E - selectin, two adhesion molecules that cooperate to optimize neutrophil recruitment (Phillipson and Kubes, 2011; Petri et al., 2008).

When UPEC invades the kidneys, cytokines secreted by tubular epithelial cells, resident dendritic cells, or tissue - resident macrophages also recruit neutrophil (Bottek et al., 2020). The chemokine receptors CXCR1 and CXCR2, highly expressed on neutrophil surfaces, enable their recruitment to kidney infection sites by CXCL1 and CXCL (Roche et al., 2007). For example, CXCL2 secreted by Ly6C - macrophages can activate MMP - 9 on circulating neutrophil surfaces, allowing them to infiltrate epithelial tissues (Schiwon et al., 2014). Research shows that in children prone to pyelonephritis, CXCR1 expression in neutrophils is lower than in age - matched controls (Gabriela et al., 1997), suggesting susceptibility to pyelonephritis may be related to impaired neutrophil recruitment ability. Additionally, CXCR2 knockout mice have a higher chance of developing pyelonephritis, renal abscesses, and renal scarring when infected with UPEC, potentially leading to sepsis. Neutrophil - deficient mice also show a similar trend, indicating that the absence of CXCR2 is closely related to delayed neutrophil recruitment and reduced bacterial killing efficiency (Svensson et al., 2011). These findings suggest that CXCL1 and CXCL2 are crucial for neutrophil recruitment to the kidneys and that neutrophil recruitment is essential for the kidneys’ anti - infective response.

Neutrophils primarily exert their anti - infection effects through three mechanisms: phagocytosis, degranulation, and the formation of neutrophil extracellular traps (NETs), which consist of DNA released from neutrophils along with proteins that aid in pathogen capture and killing (Rosales, 2018). Their mechanisms are as follows: (1) Neutrophils are potent phagocytic cells that rapidly engulf pathogens, and this phagocytic activity is enhanced by the complement system and immunoglobulin G (IgG) antibodies (Segal, 2005). (2) The cytoplasmic granules of neutrophils are critical for pathogen destruction. These granules are classified into four types: primary, secondary, tertiary, and secretory granules (Sheshachalam et al., 2014; Paige, 2006). Primary granules contain myeloperoxidase (MPO) and neutral protease G (NPG), which directly kill and digest pathogens. Secondary granules (specific granules) contain lactoferrin, which sequesters iron and copper, limiting microbial growth. Tertial granules (gelatinase granules) include matrix metalloproteinase - 9 (MMP - 9), essential for degrading the extracellular matrix and activating interleukin - 1 beta (IL - 1β). Secretory granules contain serum albumin and pre - formed cytokines (Sheshachalam et al., 2014). During degranulation, granules are released into the extracellular environment in a specific order: secretory granules, tertiary granules, secondary granules, and finally, primary granules, contributing to their bactericidal effectsHannan, 2025). However, the efficacy of these mechanisms decreases significantly in blood flow. To address this, neutrophils have evolved NETs. (3) NETs form a web - like structure of DNA released from neutrophils, coated with neutrophil proteases (like elastase), antimicrobial molecules (like histones), and other toxic components for pathogen clearance (Kolaczkowska et al., 2015; Phillipson and Kubes, 2011). These components effectively trap and kill pathogens in the bloodstream. NETs formation begins with nuclear membrane degradation and DNA release triggered by reactive oxygen species (ROS) generated by neutrophils (Lin et al., 2024; Cotzomi-Ortega et al., 2024). NETs play a crucial role in controlling invasive infections. In experiments with mice treated with exogenous DNase to degrade NETs, an increase in bacteremia incidence and bacteria escape from the skin were observed (Yipp et al., 2012). In conclusion, neutrophils, as significant innate immune cells, play a crucial role in combating urinary tract pathogens.

### 3.2 Macrophages and dendritic cells

In addition to neutrophils, macrophages and dendritic cells are also essential for the body’s defense against urinary tract pathogen invasion (**Figure 2**). When pathogens invade bladder epithelial cells, macrophages respond rapidly and show strong phagocytic abilities, limiting pathogen proliferation and spread through phagocytosis. Dendritic cells also have the capacity to phagocytose pathogens, and this ability gradually increases within 24 hours post - infection (Mass et al., 2023). However, some studies indicate that in the presence of both macrophages and dendritic cells, the phagocytic function of dendritic cells may be suppressed by macrophage (Bowyer et al., 2022). Moreover, the competition between them for phagocytosing bacteria may lead to a decrease in the antigen - presenting efficiency of dendritic cells, thus weakening the adaptive immune response (Mora-Bau et al., 2015). This implies that their interactions can affect the scale and degree of both innate and adaptive immune responses. The complex network of dendritic cells and macrophages in renal tissue can detect the state of the renal interstitium by sensing self - antigens, danger signals, as well as pathogen - associated molecular patterns (PAMPs) and damage - associated molecular patterns (DAMPs) released by bacteria in the glomeruli and renal tubules (Mariano and Ingersoll, 2020; Hayes and Abraham, 2016a, Weisheit et al., 2015; Sedin et al., 2019; Hayes and Abraham, 2016). This sentinel function enables them to coordinate TLR4 - mediated antimicrobial defense programs.

In the early stages of infection, uropathogenic Escherichia coli (UPEC) releases PAMPs and DAMPs into the urine and surrounding tissues, which can affect the resident macrophage population and the recruited Ly6C + monocyte population, promoting macrophage differentiation toward the “M2 type” (Wang et al., 2014; Davies et al., 2013; Galli et al., 2011). Tissue - resident macrophages recruit Ly6C + monocyte - derived macrophages to the bladder lamina propria by activating the NF - κB signaling pathway (Mariano et al., 2020). These macrophages, through the expression of iNOS and inflammatory cytokines such as TNF and IL - 1β, stimulate the secretion of CXCL2 from Ly6C - macrophages, facilitating the recruitment of dendritic cells and circulating neutrophils into the epithelial tissue to combat infection (Ruiz-Rosado et al., 2021; Berry et al., 2017; Schiwon et al., 2014; Tsou et al., 2007). In studies on mice with pyelonephritis, depleting Ly6C + macrophages and reducing the secretion of inflammatory cytokines and chemokines can stop the increased recruitment of circulating monocytes to the kidneys. Notably, with the reduction of monocytes, the bacterial load in the kidneys decreases, suggesting that Ly6C + macrophages derived from monocytes may have a dual role in promoting inflammation and bacterial dissemination during pyelonephritis (Ruiz-Rosado et al., 2021).

Recently, resident macrophages in the bladders of female C57BL/6 mice have been classified into two functionally distinct populations: muscle layer resident macrophages and lamina propria resident macrophages. Both populations can be supplemented by recruiting monocytes (Mariano et al., 2020). RNA sequencing analysis shows an increased expression of genes related to endocytosis and the formation of phagosomes and lysosomes in muscle layer resident macrophages. This suggests that these macrophages may have enhanced anti - inflammatory and phagocytic activities during early urinary tract infections. In contrast, lamina propria resident macrophages show high expression of chemokines and genes related to TLR signaling pathways, indicating a greater propensity for pro - inflammatory responses (Mariano et al., 2020). Notably, the pro - inflammatory capability of lamina propria resident macrophages is similar to that of recruited Ly6C + circulating monocytes in the bladder lamina propria, suggesting that Ly6C + circulating monocytes could be precursors to lamina propria resident macrophages. The latest research has demonstrated that resident macrophages can release Macrophage Extracellular Traps (METs), which are similar to neutrophil extracellular traps (NETs), for bacterial clearance (Hannan, 2025). However, there is still relatively little research on resident macrophages in the bladder, which may become a new direction for studying the anti-infective immunity of the bladder.

### 3.3 Other innate immune cells

In addition to neutrophils, macrophages, and dendritic cells, other innate cells in the urinary tract immune network also play significant roles. Mast cells (MCs), as bladder immune sentinel cells, are mainly located in the bladder lamina propria and detrusor muscle (**Figure 2**). During early infection, mast cells show notable proliferation and migration towards the infection site and can accelerate neutrophil recruitment by releasing pre - stored granule mediators from their cytoplasm (Doener et al., 2013; Siebenhaar et al., 2007; Austen, 2001; Roger et al., 2001). Research shows that mast cell absence impairs neutrophil recruitment and activation, affecting bacterial clearance (Doener et al., 2013). Moreover, inflammatory factors in cytoplasmic granules, like tumor necrosis factor, can also speed up dendritic cell recruitment and adaptive immune response formation (Shelburne et al., 2009). Additionally, mast cells are crucial for promoting homeostasis and tissue recovery after infection resolution (Choi et al., 2016). Recent studies indicate that several hours after bladder infection, mast cells express large amounts of the immunosuppressive cytokine IL - 10, corresponding to bladder epithelial cell shedding and repair (Chan et al., 2013). This suggests that mast cell - derived IL - 10 may facilitate bladder epithelial cell recovery; however, this immunosuppressive phenomenon may lead to premature elimination of adaptive immune responses, affecting the pathological process (Chan et al., 2013). Thus, mast cells have dual “pro - inflammatory” and “anti - inflammatory” roles in UTIs, and regulating these functions influences the UTI course. However, at present, the research regarding the role of mast cells in urinary tract infections (UTIs) is relatively scarce, and numerous aspects remain unresolved. For instance, how do mast cells accomplish the transition between the “pro - inflammatory” and “anti - inflammatory” states during the course of UTIs? Does the interleukin - 10 (IL - 10) secreted by them play a pivotal role? Does it exert its functions in UTIs via alternative pathways? And so forth. All these queries necessitate further in - depth investigation.

Innate lymphoid cells (ILCs) and innate - like T cells also have a role in the response to UTIs. Innate - like lymphoid cells are equivalent effector cells to T cells, residing and integrated within tissues, lacking antigen receptors on their surfaces like T and B cells (Artis and Spits, 2015; Naskar and Choi, 2024). They present and process antigens via major histocompatibility complex class II (MHC II) molecules, indirectly regulating antigen - specific T cells (Vivier et al., 2018; Naskar and Choi, 2024). Functionally, ILCs can be divided into five subgroups: natural killer (NK) cells, ILC1, ILC2, ILC3, and lymphoid tissue inducer (LTi) cells (Vivier et al., 2018; Liu et al., 2025; Huang et al., 2022). Among them, NK cells mainly reflect CD8 + T cell functions, while ILC1, ILC2, and ILC3 represent Th1, Th2, and Th17 type T cells respectively. Notably, NK cells make up about 2% of bladder immune cells in healthy juvenile mice and can kill pathogens by releasing perforin, granzymes, and pro - inflammatory cytokines (Bowyer et al., 2022). However, the relative contributions of NK cell subpopulations in the bladder are unclear. Additionally, recent studies show that ILC3s are highly enriched in mucosal tissues, can regulate Th17 cell responses and participate in innate immune responses against extracellular bacteria (Hepworth et al., 2013). This includes inducing epithelial cells to express antimicrobial peptides via IL - 22 production and enhancing antiviral proteins for INF - λ signaling to resist pathogen invasion (Vivier et al., 2018). Some studies report sex differences in ILC3 populations, with more ILC3s in normal male juvenile bladders than in females, but a more pronounced increase in females after UPEC challenge (Zychlinsky et al., 2019). This may indicate a sex - related distribution and proliferation of ILC3s, but further investigation is needed to explore links to UTI incidence and prognosis.

Mucosal - associated invariant T (MAIT) cells are a unique subset of unconventional T cells, including γδ - T cells, natural killer T (NKT) cells, and MAIT cells, found mainly in barrier tissues like the gut, skin, and lungs, where they express T cell receptors (TCR) (Ribot et al., 2021). Notably, γδ - T cells are important for eliminating bacteria from the bladder, probably due to high IL - 17 levels. Research shows that granulocyte colony - stimulating factor (G - CSF), which regulates neutrophil maturation and release, is influenced by IL - 17 - producing γδ - T cells (Ley et al., 2006). This implies a significant role of γδ - T cells in neutrophil recruitment. However, current studies have not clearly established the role of IL - 17A secreted by γδ - T cells in adaptive immune responses (Chamoun et al., 2020). MAIT cells, associated with the microbiome, are activated by recognizing riboflavin metabolites produced by bacteria, and are abundant in mucosal tissues (Gibbs et al., 2016; Booth et al., 2015; Terpstra et al., 2020). Evidence shows that MAIT cells in mouse UTI models migrate to the bladder during infection, reducing bacterial load. A study on human UTIs also found MAIT cells in patient urine (Cui et al., 2015). These suggest that MAIT cells may play a significant role in human UTIs (Dias et al., 2016). Recent research shows that MAIT cells can reside in renal tissue by expressing CD69 and CD103 (Law et al., 2019), and when activated, secrete cytokines like IL - 2, IL - 17A, and granulocyte - macrophage colony - stimulating factor (GM - CSF) (Terpstra et al., 2020). These cytokines may act as a first line of defense against pathogen invasion in the kidneys. Although the mechanisms involving MAIT cells in UTIs are under - explored, existing evidence suggests they may be critical for the body’s defense against pathogen invasion during UTIs.

## 4 Antimicrobial mechanisms of adaptive immune cells and molecules

### 4.1 T cell

Compared with the innate immune system, which rapidly responds to pathogens by recognizing pathogen - associated molecular patterns (PAMPs) or damage - associated molecular patterns (DAMPs) non - specifically, the activation and establishment of the adaptive immune system, characterized by strong specificity and immunological memory, takes a longer time (Neugent et al., 2020) (**Figure 2**). T cells and B cells, as core components of the adaptive immune system, play important roles in fighting urinary tract infections (UTIs) (Song and Deng, 2020). During a UTI, uropathogenic Escherichia coli (UPEC) adheres to urinary tract epithelial cells and invades tissues, triggering a series of signaling pathways and host defense mechanisms that activate immune cells, including T cells, to enter the infection site and combat pathogen invasion (Isaacson et al., 2017). Research shows that after a UTI begins, the number of CD4 + T cells in the bladders of female mice significantly increases (Scharff et al., 2019), with various subpopulations detected, including Th1, Th2, Th17 cells, and regulatory T cells (Tregs) (Mariano et al., 2020). The emergence of Tregs reflects the host’s downregulation of immune responses to maintain the integrity of the urinary tract epithelium (Yu et al., 2019). Moreover, recent studies have revealed that T cells activated and migrated to the bladder after infection have limited ability to eliminate bacteria (Wu et al., 2020; Li et al., 2019; O’Brien et al., 2018); these T cells are more likely to secrete IL - 4, which promotes tissue repair, rather than IFN - γ, which is associated with bacterial clearance, suggesting a preferential differentiation towards the Th2 phenotype rather than the Th1 phenotype (Wu et al., 2020). Additionally, about 50% of CD11c + CD301b + dendritic cells in the bladder express OX40L, a molecule that preferentially polarizes CD4 + T cells towards the Th2 type (Schuijs et al., 2019; Wu et al., 2020). This cell differentiation tendency helps protect deeper bladder tissues from the harmful effects of salts, urea, and other substances in the urine when the epithelial cell shedding program is activated, but it also reduces the ability to eliminate bacteria in the bladder. This guided T cell differentiation indicates that the host balances antibacterial immune responses and repair processes when pathogens invade, aiming to resist pathogen entry and further infection with minimal damage.

The specific mechanisms of T cell - mediated anti - infective protection during urinary tract infections remain unclear. Activated Th1 and Th17 cells after infection may play a role in bacterial clearance by secreting cytokines IFN - γ and IL - 17, thereby further activating macrophages and CD8 + T cells or accelerating neutrophil recruitment (Mariano et al., 2020; Bowyer et al., 2022). Overall, research on adaptive T cells in the bladder is still in its early stages, and many related mechanisms remain undefined. As research on bladder immunity in urinary tract infections progresses, it is crucial to focus on the roles of different T cell subtypes, especially regulatory T cells, in anti - infective immunity in the bladder. Elucidating the mechanisms that maintain the delicate balance between limiting immune responses and promoting bacterial clearance will be crucial for future research. A deeper understanding of the roles of T cells in resisting urinary tract infections and the differentiation of memory T cells will not only help reveal the diversity of urinary tract immune responses but also assist in developing more effective treatment strategies and preventive vaccines for urinary tract infections.

### 4.2 Tissue-resident memory T cell

Tissue - resident memory T (Trm) cells, a subset of memory T cells, consist of unique, non - migratory cell populations that permanently reside in organs like the skin, intestines, and lungs (Gebhardt et al., 2009) (**Figure 2**). They play a crucial role in providing long - term immune protection (Longet and Paul, 2023).

In one study, researchers used lymphocyte - depleting antibodies to deplete systemic circulating T cells to verify the role of tissue - resident memory T cells in resisting recurrent urinary tract infections. The results showed that treated mice had similar anti - infection responses as untreated mice, with a sustained increase in T cell numbers detected in the bladders of treated mice (Rousseau et al., 2023). This finding emphasizes the important protective role of tissue - resident memory T cells in establishing adaptive immunity in the bladder. Moreover, research has found that CD4 + tissue - resident memory T cells are more abundant than CD8 + tissue - resident memory T cells in healthy human kidneys (van der Putten et al., 2021; Krebs et al., 2020). However, in patients with glomerulonephriti, the amounts of CD4 + and CD8 + tissue - resident memory T cells in the kidneys are nearly equal (Lisboa et al., 2020; Krebs et al., 2016). This indicates that during glomerulonephritis, there may be a greater increase in the number of CD8 + tissue - resident memory T cells in the human kidneys, although the reasons for this disproportional proliferation remain unclear. In a mouse model of pyeloneinitis, the number of renal tissue - resident memory T cells also showed a significant increase (Krebs et al., 2020). However, there is currently no data to support whether CD4 + and CD8 + tissue - resident memory T cells show different degrees of increase in this model. It is unclear how renal tissue - resident memory T cells adapt to their environment; however, studies have shown that liver tissue - resident memory T cells upregulate hypoxia - inducible factor pathways to adapt to the hypoxic conditions in the liver (Kim et al., 2020). Similarly, renal tissue - resident memory T cells in hypoxic environments may adapt through similar mechanisms and contribute to anti - infective immunity, providing a new research direction for studying the adaptive immune mechanisms within the bladder during urinary tract infections.

### 4.3 B cell

After infection onset, dendritic cells and macrophages present antigens to B cell germinal centers by phagocytosing pathogens, leading to B cell activation. Activated B cells can differentiate into either effector B cells or memory B cells (**Figure 2**). Effector B cells (plasma cells) combat pathogens by producing antibodies, while memory B cells can quickly initiate adaptive immune responses upon re - exposure to the same pathogen (Seifert and Küppers, 2016; Hawas et al., 2023). Additionally, after B cell activation, these cells can influence T cell activation through antigen presentation, provision of co - stimulatory factors, and secretion of cytokine (Petersone et al., 2018; Bishop, 2016). Among these, the co - stimulatory factor OX40 ligand (OX40L) is a crucial regulatory molecule for the proliferation and survival of T cells, promoting the differentiation of CD4 + T cells towards the Th2 phenotype (Rogers et al., 2001; Linton et al., 2003). Furthermore, recent research on tissue - resident B cells has categorized them into two subsets: B - 1 and B - 2 cells. Notably, B - 1 cells, which are enriched in the pleural and peritoneal cavities, have also been found in damaged and infected skin, lungs, and kidneys (Suchanek et al., 2023; Denton et al., 2019; Stark et al., 2018). These B - 1 cells form a significant part of the pool of tissue - resident B cells in these organs [137]. Studies have shown a negative correlation between the number of tissue - resident B cells in the kidneys and susceptibility to urinary tract infections (Suchanek et al., 2023). Moreover, the expression of chemokine transcripts for neutrophil and monocyte recruitment is elevated in the kidneys of B cell - deficient mice (Suchanek et al., 2023), suggesting that tissue - resident B cells may inhibit renal antibacterial immunity and affect the recruitment of circulating immune cells to infected organs.

Single - cell sequencing results also reveal the presence of plasma cells producing secretory IgA in the urethral lamina propria of healthy mice, with secretion levels increasing with age (Ligon et al., 2020). It is unclear whether the observed changes in cell proportion and secretion levels contribute to the increased prevalence of urinary tract infections in the elderly or if they reflect a response to repeated infections. However, the role of secretory IgA in bladder anti - infection remains uncertain. Thus, although some studies have investigated the role of B cells in urinary tract infection defenses, their exact protective roles and mechanisms need further investigation to clarify.

## 5 Conclusions and perspectives

This review mainly summarizes the functions and mechanisms of innate and adaptive immune cells in the body’s defense against urinary tract infections (UTIs). In the study of urinary tract anti - infective immunity, neutrophils, dendritic cells, macrophages, and other innate immune cells cooperate to form an effective immune network. Although we have obtained initial insights into the crucial role of bladder immunity in UTIs, there are still many research gaps. Current studies by domestic and international scholars mainly focus on the quantity and types of immune cells related to the body’s anti – infective immune mechanisms, paying less attention to changes in the bladder mucosal microenvironment and at the molecular level. Moreover, our understanding of the adaptive immune mechanisms in the bladder is insufficient and requires more detailed studies to clarify the complex regulatory networks and potential mechanisms of action (**Table 1**).

**Table 1:** Controversies or unresolved questions in the immune mechanisms of the urinary tract against infections.

Cell type	Problems to be solved/Controversial issues
Macrophages	What is the precursor of resident macrophages? What are the bactericidal mechanisms other than the use of METs?
Mast cells	How does the mast cell switch between “pro - inflammation” and “anti - inflammation” during UTI? Does the IL-10 released by it play a decisive role? Does it play a role in UTI through other pathways?
Innate lymphoid cells	The contribution of innate lymphoid cells (ILCs) to the bladder-resident cell population in the fight against urinary tract infection (UTI) remains unclear, and the specific mechanism of their action is also unknown. Whether there is a gender difference in the distribution of ILCs still needs to be further verified.
γδ-T cells	The role of γδ-T cells in the recruitment of neutrophils remains unclear. The role of IL-17A secreted by γδ-T cells in the adaptive immune response is also indistinct. There is a paucity of research associated with γδ-T cells.
T cells(Th1, Th2, Th17, and Treg cells)	The specific differentiation mechanism of memory T cells remains unclear. The specific mechanisms underlying the balance between “pro-inflammatory” and “anti-inflammatory” states among Th1, Th2, Th17, and Treg cells remain to be further clarified.
Tissue-resident memory T cells	The significance of the unequal proliferation of CD4+ and CD8+ tissue-resident memory T cells (Trm) during urinary tract infection (UTI) remains unclear. The impact of hypoxia on the functions of Trm cells in the bladder is also unknown.
B cells	The specific role of plasma cells that produce secretory immunoglobulin A (sIgA) during urinary tract infection (UTI) remains unclear. The specific protective role of B cells in the urinary tract against infection is not well-defined.

In exploring the functional mechanisms of adaptive immune cells, tissue - resident memory T cells have become a new focus. There is an urgent need to further understand the mechanisms of how tissue - resident memory T cells are established and maintained on mucosal surfaces and to know about the potential migration of these cells. Additionally, mucosa - associated invariant T cells have been found to have anti - infective effects in the kidneys through cytokine secretion, but their location during bladder inflammation is unclear, which provides a potential new research direction for anti - urinary tract infection immunity. B cells, as a part of adaptive immunity, seem to have bacteriostatic effects different from those of T cells, which requires further investigation. Some evidence also suggests that when initiating anti - infective immunity, the body tends to favor the subtype differentiation of immune cells towards “tissue repair” rather than “bacterial clearance”. This indicates that the body may prioritize minimizing tissue damage over attacking pathogens, aiming to achieve a dynamic balance with pathogens to relieve inflammatory symptoms and ensure normal physiological functions. These studies emphasize the complex relationships among immune cells, as different immune cells usually work together in response to pathogen invasion. A more comprehensive and holistic view is necessary to understand these immune cells.

Exploring novel prevention and treatment methods beyond traditional antibiotics is crucial for managing urinary tract infections (UTIs), particularly those caused by multidrug-resistant bacteria. Recent research has found that T cells in peripheral circulation do not influence the outcomes of urinary tract infections, while tissue-resident memory (TRM) T cells in the bladder play a critical role. This new finding is significant for the development of vaccines and immunotherapeutic strategies for urinary tract infections. Additionally, the role of γδ T cells that produce IL-17 in combating urinary tract infections is also noteworthy. Antibodies IgA and IgG can be detected in the blood and urine of UTI patients; however, the precise role of these antibodies in the risk assessment of UTIs remains uncertain and requires further investigation.

Overall, future research will concentrate on a deeper understanding of the mechanisms of immune cells such as tissue - resident memory T cells, mucosal - associated invariant T cells, and B cells, which are not yet clearly defined in bladder immunity. This understanding will guide the design of innovative vaccines to strengthen the immune system’s defense against urinary tract infections, providing more effective methods for clinical treatment and prevention.
